# Expression Profiles of tRNA-Derived Small RNAs and Their Potential Roles in Primary Nasopharyngeal Carcinoma

**DOI:** 10.3389/fmolb.2021.780621

**Published:** 2021-12-20

**Authors:** Zhaoyi Lu, Kai Su, Xiaomin Wang, Mingjie Zhang, Shiyin Ma, Hui Li, Yuanzheng Qiu

**Affiliations:** ^1^ Department of Otolaryngology-Head and Neck Surgery, Xiangya Hospital, Central South University, Changsha, China; ^2^ Department of Otolaryngology-Head and Neck Surgery, The First Affiliated Hospital of Bengbu Medical College, Bengbu, China

**Keywords:** nasopharyngeal carcinoma, tRNA-derived small RNAs, tRNA-related fragments, TRNA halves, non-coding RNAs, bioinformatics

## Abstract

**Introduction:** tRNA-derived small RNAs (tsRNAs), a class of small non-coding RNAs, are divided into two categories: tRNA-related fragments (tRFs) and tRNA halves (tiRNAs). Abnormal expression of tsRNAs has been found in diverse cancers, which indicates that further understanding of the function of tsRNAs will help identify new biomarkers and potential therapeutic targets. Until now, the underlying roles of tsRNAs in primary nasopharyngeal carcinoma (NPC) are still unknown.

**Methods:** tRF and tiRNA sequencing was performed on four pairs of NPC tissues and healthy controls. Thirty pairs of NPC samples were used for quantitative real-time polymerase chain reaction (qRT-PCR) verification, and the ROC analysis was used to evaluate the diagnostic efficiency initially. Target prediction and bioinformatics analysis of validated tRFs and tiRNAs were conducted to explore the mechanisms of tsRNAs in NPC’s pathogenesis.

**Results:** A total of 158 differentially expressed tRFs and tiRNAs were identified, of which 88 are upregulated and 70 are downregulated in NPC. Three validated tRFs in the results of qRT-PCR were consistent with the sequencing data: two upregulations (tRF-1:28-Val-CAC-2 and tRF-1:24-Ser-CGA-1-M3) and one downregulation (tRF-55:76-Arg-ACG-1-M2). The GO and KEGG pathway enrichment analysis showed that the potential target genes of validated tRFs are widely enriched in cancer pathways. The related modules may play an essential role in the pathogenesis of NPC.

**Conclusions:** The tsRNAs may become a novel class of biological diagnostic indicators and possible targets for NPC.

## Introduction

Nasopharyngeal carcinoma (NPC) is a malignant epithelial tumor that occurs on the top and side walls of the nasopharynx (1). The incidence of NPC in head and neck tumors is the highest in southern China (>20/100,000), while it is extremely low in European and American countries (<1/100,000), with women 2–3 times more likely to be affected than men (Jemal et al., 2011; Zhang et al., 2015; Chen et al., 2019). The etiology of NPC is complicated, including Epstein–Barr virus (EBV) infection, genetic factors, and the geographical environment (Wei and Sham, 2005). Due to the hidden location and unobvious early symptoms of NPC, the clinical diagnosis occurs mostly in the advanced stage. High incidence and fatal metastasis are the leading causes of death from NPC. With the development of radiotherapy and chemotherapy, local metastasis and recurrence are still prone to occurring (Chan, 1990; Chen et al., 2014). Therefore, it is vital to explore the pathogenesis of NPC and discover new diagnostic and therapeutic targets (Tang et al., 2018).

With the rapid development of RNA sequencing, new ncRNAs are constantly being discovered; in the biological process, ncRNAs usually play different roles and have always been a hot spot in the research on cancer and other diseases. According to their size, ncRNAs can be divided into three categories: the long non-coding RNAs (lncRNAs) that are longer than 200 nucleotides (nt), the medium ncRNAs that are 40 nt–200 nt, and the small ncRNAs that are less than 40 nt (Zeng et al., 2020). Among small ncRNAs, in addition to siRNA, microRNA, and piRNA, there are also tRNA-derived small RNAs (tsRNAs, with lengths of 14 nt−40 nt) that have recently gained attention. In the 1970s, researchers thought that tsRNAs were products of random degradation of tRNA (Borek et al., 1977; Speer et al., 1979). With continuous research in the past 20 years, it has been found that tRNAs can be further accurately sheared to tsRNAs and play unique and diverse biological roles under different physiological and pathological conditions, including regulating protein translation, gene expression, retrotransposon regulation, chromatin remodeling, epigenetic modifications, stress response, and immune signals (Zhu et al., 2019a; Cao et al., 2020; Kim et al., 2020). tsRNAs fell into two major categories: tRNA-related fragments (tRFs) derived from mature or precursor tRNAs and tRNA halves (tiRNAs) generated by specific cleavage in the anticodon loops of mature tRNAs (Shen et al., 2018). tRFs and tiRNAs are classified into five types depending on where the map on the precursor or mature tRNA transcript digested by angiogenin (ANG), dicer, or other RNases: tRF-5, tRF-3, tRF-1, tRF-2, and tiRNA (Kumar et al., 2016).

Studies have shown that tRFs and tiRNAs are involved in regulating cancer cell growth, proliferation, metastasis, and progression and can even initiate viral reverse transcriptase (Ruggero et al., 2014; Huang et al., 2018; Li et al., 2018; Yu et al., 2020). Its diverse biological effects have been verified in breast cancer, colorectal cancer, ovarian cancer, lung cancer, prostate cancer, and chronic lymphocytic leukemia (CLL), which provided a basis for tRFs and tiRNAs as diagnostic and prognostic biomarkers (Shao et al., 2017; Zhu et al., 2019a; Veneziano et al., 2019; Shen et al., 2020; Zhang et al., 2020). At the same time, it is also worthy of further exploration as a potential therapeutic target for cancer (Kim et al., 2020). At present, both endogenous regulation of tsRNAs and exogenous addition of tsRNAs have achieved preliminary results. Increased LeuCAG3′tsRNA upregulates the number of ribosomes, promoting the proliferation of hepatocellular carcinoma cells (Kim et al., 2017) and the addition of exogenous tsRNAs derived from tRNA^GluYTC^ to suppress breast cancer cell metastasis *via* destabilizing oncogenic transcripts by competing for binding with the YBX1 protein (Goodarzi et al., 2015). Aristeidis et al. also reported that tRFs (nuclear tRNA^HisGTG^ and tRNA^AlaTGC^ and the mitochondrial tRNA^GluTTC^) could stabilize oncogenes through a binding matched motif of HuR in triple-negative breast cancer (Telonis and Rigoutsos, 2018). However, the research on tsRNA profiles in NPC is still blank, and the detailed mechanisms governing NPC pathogenesis remain unknown. We speculate that the abnormally expressed tsRNAs are likely to be a specific regulatory molecule for NPC, playing an essential role in the tumorigenesis and development. This article is the first to carry out tRF and tiRNA sequencing of NPC to screen and verify NPC-specific biomarkers. Besides, we constructed a co-expression network for the validated tRFs and performed further bioinformatic analysis. Finally, we analyzed and explored the potential molecular mechanisms of tRFs and tiRNAs in NPC. This study provided new insights for the diagnosis and treatment of NPC in clinical application.

## Materials and Methods

### Clinical Specimens

The study was approved by the Clinical Research Ethics Committee of The First Affiliated Hospital of Bengbu Medical College. Written informed consent was obtained from all-volunteer patients. Fresh NPC tissue specimens (n = 4) and mucosal tissue specimens (n = 4) were acquired from primary NPC and nasopharyngitis patients *via* transnasal endoscopic biopsy for tRF and tiRNA sequencing. Then, a total of 30 unmatched specimens, containing the samples for sequencing, were used to validate the tsRNA expression by qRT-PCR. All patients were newly diagnosed with NPC without any radiotherapy or chemotherapy and had no history of other tumors, and the samples were confirmed by histopathological diagnosis. The TNM staging method adopted the eighth edition of the Union for International Cancer Control (UICC) and the American Joint Committee on Cancer (AJCC). All samples were stored in a liquid nitrogen tank (−180°) immediately after being separated.

### Total RNA Extraction and tRF and tiRNA Sequencing

Total RNA was extracted from frozen samples according to the manufacturer’s instructions on TRIzol (Invitrogen, CA, United States). Before the sequencing experiment, we checked each RNA sample’s integrity and quantity using agarose gel electrophoresis and a Nanodrop™ instrument (ND-1000, Thermo Fisher Scientific, DE, United States). tsRNAs are heavily decorated by RNA modifications that interfere with small RNA-seq library construction. We do the following treatments using a rtStar™ tRF and tiRNA Pretreatment Kit (AS-FS-005, Arraystar Inc., MD, United States) before library preparation for total RNA samples: 3′-aminoacyl (charged) deacylation to 3′-OH for 3′adaptor ligation, 3′-cP (2′,3′-cyclic phosphate) removal to 3′-OH for 3′adaptor ligation, 5′-OH (hydroxyl group) phosphorylation to 5′-P for 5′-adaptor ligation, and m1A and m3C demethylation for efficient reverse transcription. cDNA was then synthesized and amplified using Illumina’s proprietary RT primers and amplification primers. Subsequently, ∼134–160 bp PCR amplified fragments were extracted and purified from the PAGE gel. The libraries are qualified and quantified absolutely using an Agilent BioAnalyzer 2100 (Agilent Inc., MD, United States). For standard small RNA sequencing on the Illumina NextSeq 500 system (NextSeq 500/550 V2 kit, Illumina, CA, United States), the sequencing type is 50 bp single read.

### tRF and tiRNA Data Analysis

Comprehensive data processing and statistical analyses are following the Arraystar tRFs and tiRNAs-seq data analysis workflow. The sequencing quality is examined by FastQC (http://www.bioinformatics.babraham.ac.uk/projects/fastqc/) and trimmed reads (pass Illumina quality filter, trimmed 5′,3′-adaptor bases by cutadapt (Kechin et al., 2017)) are aligned, allowing for one mismatch only to the mature tRNA sequences, and then reads that do not map are aligned, allowing for one mismatch only to precursor tRNA sequences with bowtie software (Langmead et al., 2009). Based on alignment statistical analysis (mapping ratio, read length, and fragment sequence bias), we determine whether the results can be used for subsequent data analysis. The abundance of tRFs and tiRNAs are evaluated using their sequencing counts and are normalized as counts per million (CPM) of total aligned reads. The tRFs and tiRNAs differentially expressed are screened based on the edgeR package (version 3.20.9) in the statistical R program (version 3.6.0). All visual analysis plots were outputted using the R package and Microsoft Office Excel.

### Validation With Quantitative Real-Time PCR

The total RNA extraction method of tissue samples is the same as above. After dissolving with diethylpyrocarbonate water, the OD 260/280 absorbance ratios are checked and stored in a −80° refrigerator. The top five upregulated and top five downregulated tsRNAs that met specific primer design requirements were selected for verification from the differential sequencing results. The universal primer of U6 snRNA (Ribobio, China) was used as an inner reference. The Bulge–Loop miRNA Starter Kit (Ribobio, China) was adopted in the real-time quantitative polymerase chain reaction (RT-qPCR). Each indicator is supplemented with specific stem-loop RT primers to ensure the sensitivity and specificity of the reverse transcription (RT). We configure the RT reaction system on ice according to the manufacturer’s protocol, the conditions of which are 42°C, 60 min and 70°C, 10 min. The qPCR reaction was detected with SYBR Green dye using the QuantStudio™ 7 Flex Real-Time PCR System (Applied Biosystems, CA, United States), the conditions of which were 95°C, 10 min, followed by 40 cycles of 95°C, 2 s and 60°C, 34 s, using the instrument default program for melting curve analysis. The relative tRF and tiRNA expression levels were calculated using the 2^-△Ct^ method and were normalized with a control (U6 as an endogenous control for small nuclear RNA, ΔCt = Ct tRFs and tiRNAs – Ct U6). The reactions were repeated in triplicate.

### Bioinformatic Analysis

In order to explore the potential target genes of validated tRFs, we filtrated the database of TargetScan (http://www.targetscan.org/vert_72/) and Miranda (http://www.microrna.org/microrna/) with the criteria of energy < –10, structure >140, and context < −0.10 (Enright et al., 2003; Garcia et al., 2011). The Gene Ontology (GO) project provides a controlled vocabulary to describe gene and gene product attributes in any organism. The ontology covers three domains: Biological Process (BP), Cellular Component (CC), and Molecular Function (MF). Pathway analysis is a functional analysis mapping gene to KEGG pathways. We use the GO knowledge base (http://www.geneontology.org/) and the KEGG database (https://www.kegg.jp/) to perform GO analysis and pathway analysis on the potential target genes of validated tRFs. Using Fisher’s exact test to evaluate the results, *p*-value < 0.05 is considered meaningful.

### Statistical Analysis

The statistical analysis software are SPSS version 22.0 (SPSS Inc., Chicago, United States) and GraphPad Prism 7 (GraphPad Software, California, United States). The results of qRT-PCR were presented as the mean ± standard error of the mean. The difference of the results was evaluated by two-tailed Student’s *t*-test and two-way ANOVA between groups, and a *p*-value <0.05 was assumed to be statistically significant. The receiver operating characteristic (ROC) curve was used to evaluate the effectiveness of validated tRFs in the diagnosis of NPC by specificity, sensitivity, and the area under the curve (AUC). AUC values of 0.5 and 1.0 predict the random and excellent accuracy of the diagnosability, respectively.

## Results

### tRF and tiRNA Expression Profile in Primary NPC

**FIGURE 1:**
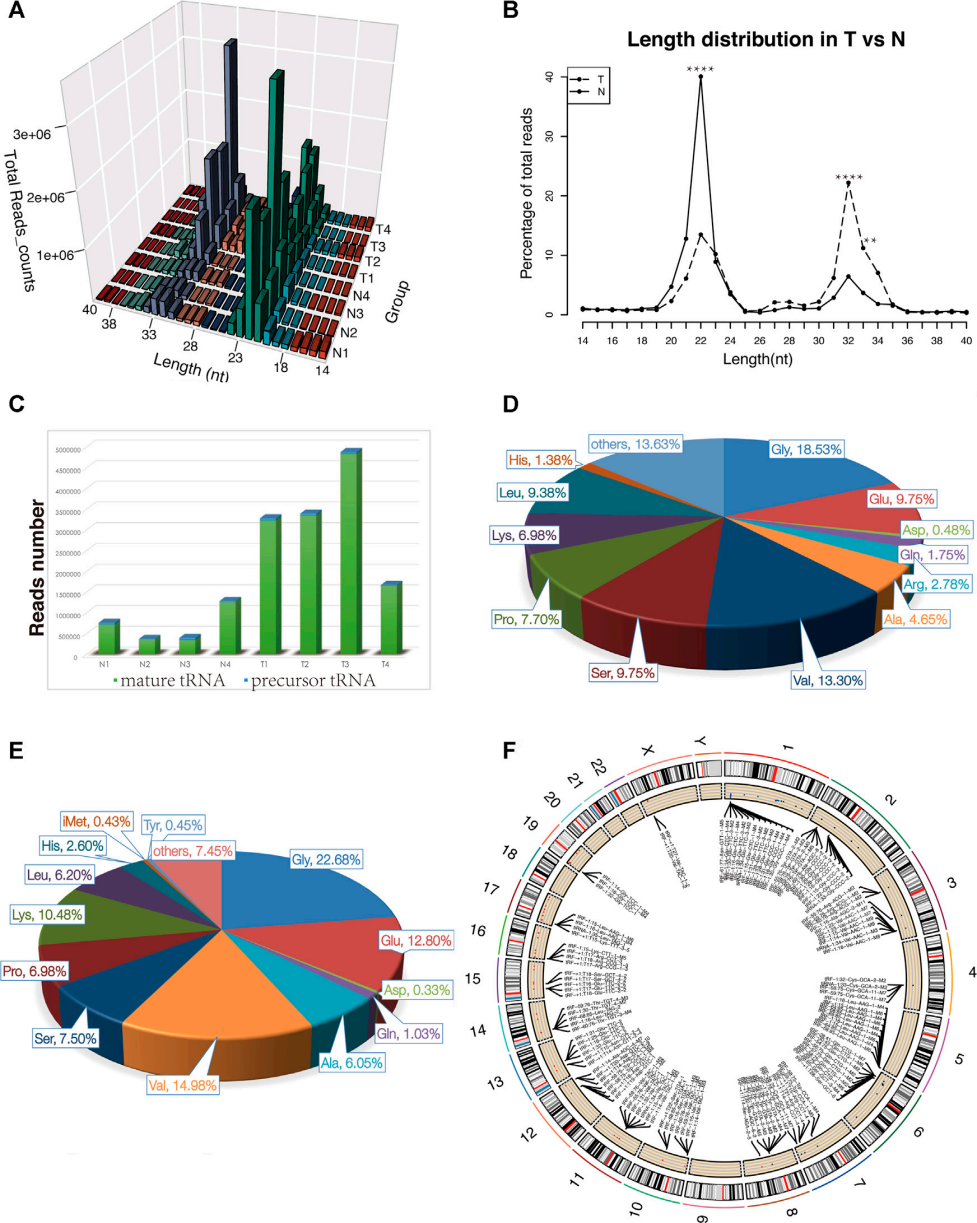
Characteristics of the detected tsRNAs in nasopharyngeal carcinoma and healthy controls. **(A)** Length distribution of the detected tsRNAs. *x*-axis, the length of the detected tsRNAs; *y*-axis, the abundance of tsRNAs classified by different lengths. **(B)** Length of tsRNAs in NPC tumor tissues and matched normal tissues mainly ranged from 14 to 40 nt **p < 0.01, ****p < 0.0001. **(C)** Reads number of the source of tRFs and tiRNAs (mature tRNA and precursor tRNA). **(D–E)** Distribution of source tRNAs in healthy controls and nasopharyngeal carcinoma. **(F)** Circos plot of detected tsRNAs on human chromosomes (visualize only part of the data). Abbreviations: T, tumor specimens; N, normal specimens.

**FIGURE 2:**
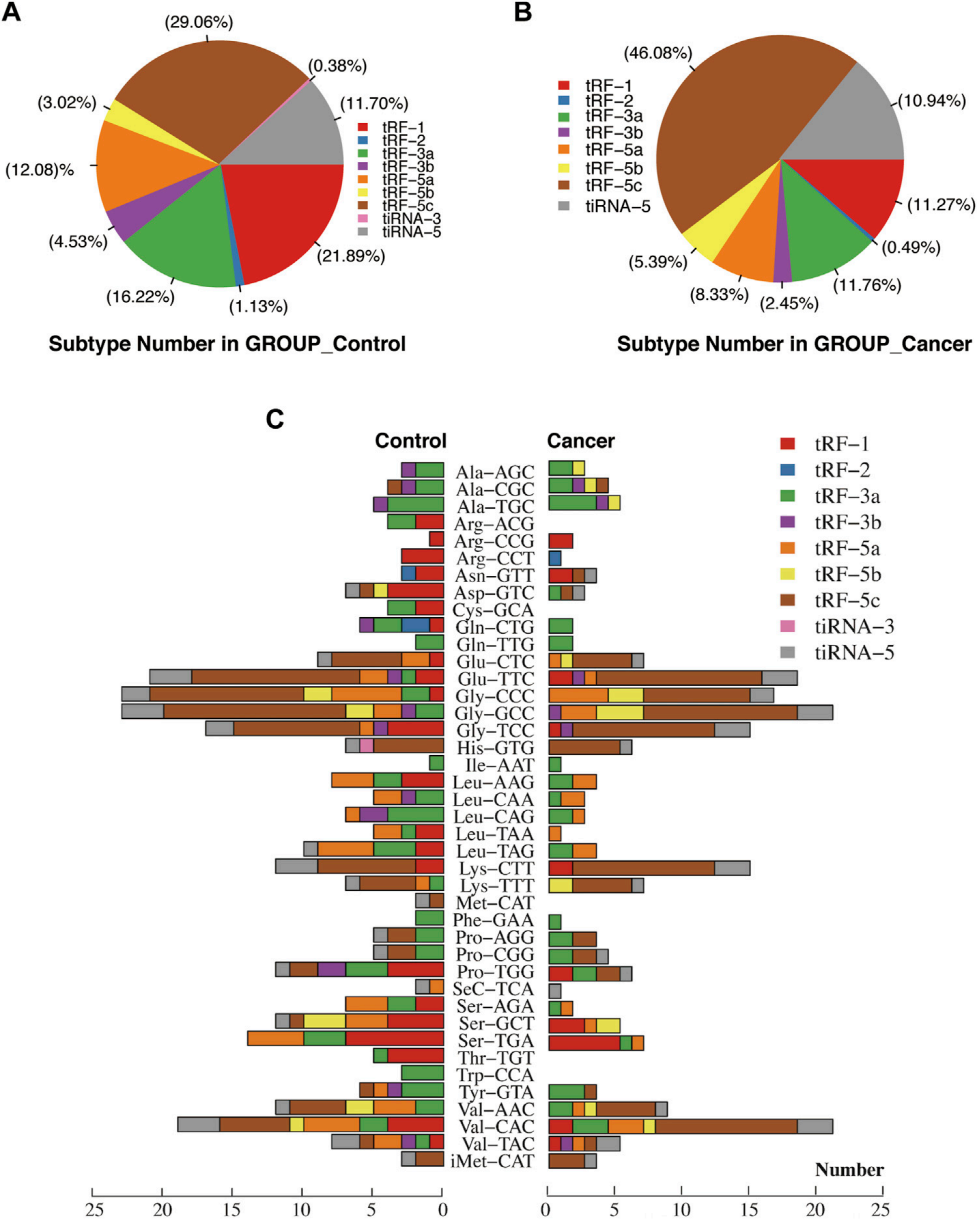
Proportions of subtype tRFs and tiRNAs against tRNA isodecoders between healthy controls and nasopharyngeal carcinoma. **(A)** Proportions of tRF-1, tRF-2, tRF-3a, tRF-3b, tRF-5a, tRF-5b, tRF-5c, tiRNA-3, and tiRNA-5 in healthy controls. **(B)** Proportions of tRF-1, tRF-2, tRF-3a, tRF-3b, tRF-5a, tRF-5b, tRF-5c, and tiRNA-5 in nasopharyngeal carcinoma. **(C)** Stacked plot for all subtypes of tsRNAs between two groups clustering by the anticodon of the tRNAs. The *X*-axis represents the number of all subtype tsRNAs derived from the same anticodon tRNA, and the *Y*-axis shows the tRNAs with the same anticodon. The bar with color represents the number of each subtype of tsRNAs. Red, blue, green, purple, orange, yellow, and brown represent the subtypes of tRFs. Pink and gray represent the two subtypes of tiRNAs.

For the first time, we performed tRF and tiRNA sequencing on primary NPC specimens. The sequence read length distribution is shown in Figure 1A. The variance analysis showed significant differences between groups at 22, 32, and 33 nt (Figure1B). The read number of the source of tRFs and tiRNAs (mature tRNA and precursor tRNA) in NPC samples was significantly higher than that of the control group (Figure 1C). Among the source tRNAs, we found that tRNAs-Gly and tRNAs-Val were most prevalent, comprising 18.5 and 13.3% in the control group (Figure 1D) and 22.7 and 15.0% in the NPC group, respectively (Figure 1E). The tRFs and tiRNAs shared the same anticodon that may be derived from different tRNA precursors. The number of subtypes of tRFs and tiRNAs against tRNA isodecoders between groups is shown in Figures 2A,B. In terms of numbers, the tRF-1 increased significantly (*p* = 0.0038, data not shown) in the control group, while the tRF-5c markedly rose in the NPC group (*p* = 0.0013, data not shown). We found that Arg-ACG, Cys-GCA, Met-CAT, Thr-TGT, and Trp-CCA were absent in the NPC group (Figure 2C). Except for chromosomes 9, 18, 20, 21, 22, and Y, tRF and tiRNA are widely distributed in most chromosomes (Figure 1F).

### Differentially Expressed tRFs and tiRNAs in Primary NPC

**FIGURE 3:**
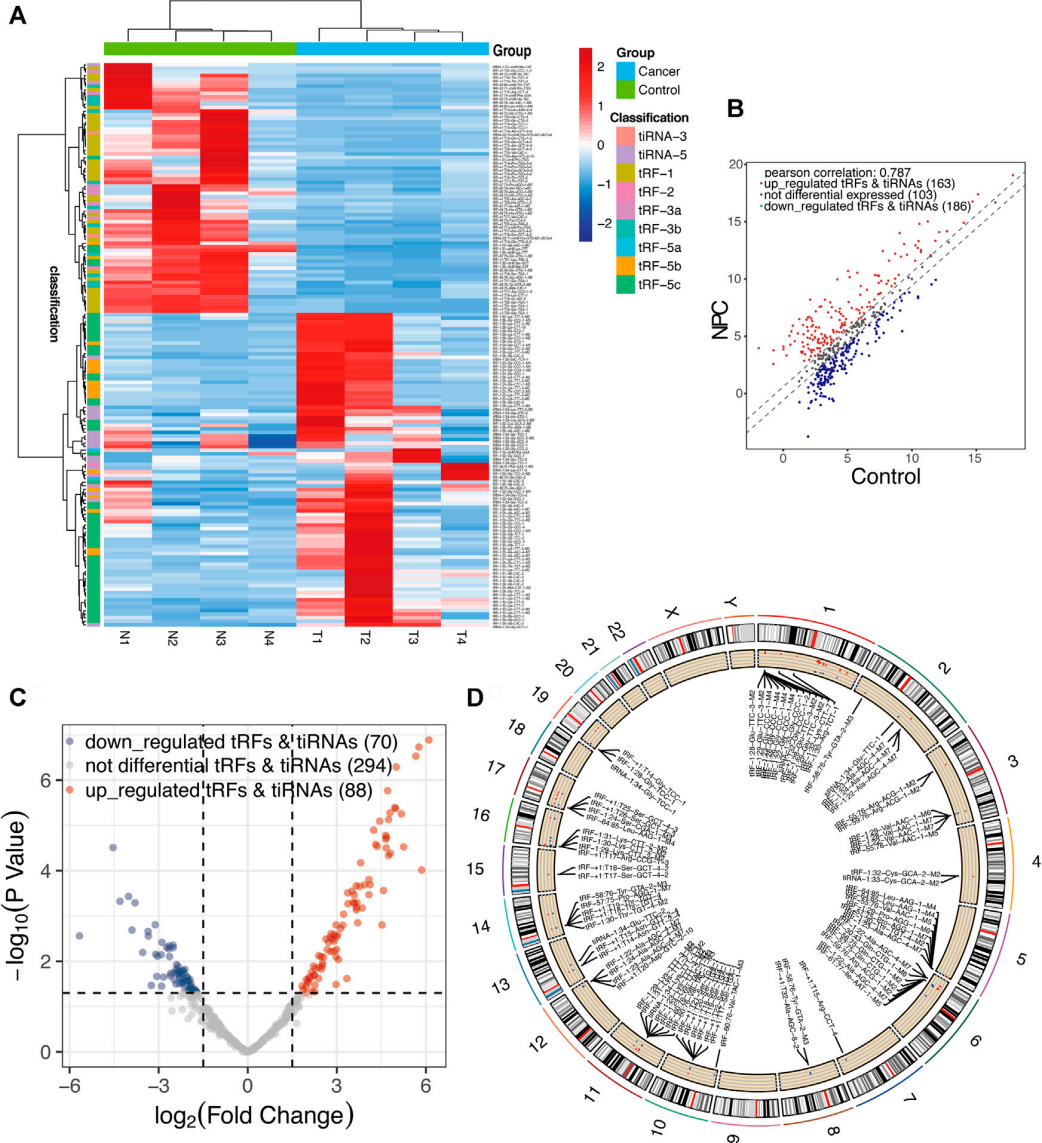
Expression profiles of all aberrantly expressed tRFs/tiRNA sequencing data in nasopharyngeal carcinoma and healthy controls. **(A)** Expression heatmap of all aberrantly expressed tRFs/tiRNAs. **(B)** Scatter plot between two groups. CPM values of all tRFs and tiRNAs are plotted. The values of the *X-* and *Y*-axes in the scatter plot are the averaged CPM values of each group (log2 scaled). tRFs and tiRNAs above the top line (red dots, upregulation) or below the bottom line (blue dots, downregulation) indicate more than 1.5-fold change between two compared groups. Gray dots indicate non-differentially expressed tRFs and tiRNAs. **(C)** Volcano plot presents differential expression between the two groups. The values of the *X-* and *Y*-axes in the volcano plot are log2 transformed fold change and -log10 transformed *p*-values between the two groups, respectively. Red/blue circles indicate statistically significant differentially expressed tRFs and tiRNAs with a fold change of no less than 1.5 and *p*-value ⩽ 0.05 (red, upregulated; blue, downregulated). Gray circles indicate non-differentially expressed tRFs and tiRNAs, with FC and/or q-value that are not meeting the cut-off thresholds. **(D)** Circos plot of all differentially expressed tsRNAs on human chromosomes (visualize only part of the data).

A total of nearly 1.75 gigabytes (Gb) of sequencing data were obtained from the four pairs of samples. A total of 452 tRFs and tiRNAs were detected, of which 373 tRFs and tiRNAs were not collected by the tRFdb database (Kumar et al., 2015) (Supplementary File S1). We used a visual method, hierarchical clustering, to analyze tRF and tiRNA expression data (Figure 3A). The tRF-5c cluster was highly expressed in the NPC group, and tRF-1 was highly expressed in the control group, consistent with the subtype tRF and tiRNA analysis results. The tRFs and tiRNAs with a fold change of no less than 1.5 and *p*-value ⩽ 0.05 are regarded as statistically significant differentially expressed genes. The scatter plot showed the tRF and tiRNA expression variation between the two groups of samples (Figure 3B). A total of 158 differential tRFs and tiRNAs were visualized by volcano plots, of which 88 are upregulated and 70 are downregulated (Figure 3C). Table 1 shows significant up- and downregulation of tRFs and tiRNAs in primary NPC vs. healthy controls. For the top 15 upregulated tsRNAs, the linear fold change varied from 68.6- to 25.5-fold. For the top 15 downregulated tsRNAs in NPC, the linear fold change ranged from 50.8- to 7.5-fold. The differentially expressed tRFs and tiRNAs were distributed by the circos plot on human chromosomes (Figure 3D).

**TABLE 1:** Top 15 significantly differentially expressed tRNA-derived small RNAs.

*tRF ID*	Fragment sequence, 5′-3′	nt	Type	log2FC	*p*_Value
tRF-1:28-Val-CAC-2	GCT​TCT​GTA​GTG​TAG​TGG​TTA​TCA​CGT​T	28	tRF-5c	6.100773855	1.30446E-07
tRF-1:24-Ser-CGA-1-M3	GCT​GTG​ATG​GCC​GAG​TGG​TTA​AGG	24	tRF-5b	5.857283971	9.62489E-05
tRF-1:29-Lys-CTT-1-M2	GCC​CGG​CTA​GCT​CAG​TCG​GTA​GAG​CAT​GG	29	tRF-5c	5.763858615	1.86215E-07
tRF-1:29-Lys-CTT-2-M2	GCC​CGG​CTA​GCT​CAG​TCG​GTA​GAG​CAT​GA	29	tRF-5c	5.662221668	2.96309E-07
tRF-1:24-Lys-TTT-3-M2	GCC​CGG​ATA​GCT​CAG​TCG​GTA​GAG	24	tRF-5b	5.248646873	2.97073E-05
tRF-1:30-Val-CAC-2	GCT​TCT​GTA​GTG​TAG​TGG​TTA​TCA​CGT​TCG	30	tRF-5c	5.177271145	5.54834E-06
tRF-1:30-Lys-CTT-2-M2	GCC​CGG​CTA​GCT​CAG​TCG​GTA​GAG​CAT​GAG	30	tRF-5c	5.003910166	5.11953E-07
tRF-1:28-Glu-CTC-1-M2	TCC​CTG​GTG​GTC​TAG​TGG​TTA​GGA​TTC​G	28	tRF-5c	4.976882067	4.24157E-06
tRF-1:28-Val-CAC-3	GTT​TCC​GTA​GTG​TAG​CGG​TTA​TCA​CAT​T	28	tRF-5c	4.952417008	3.99982E-06
tiRNA-1:33-Cys-GCA-2-M2	GGG​GGT​ATA​GCT​CAG​TGG​TAG​AGC​ATT​TGA​CTG	33	tiRNA-5	4.805270206	4.11876E-05
tRF-1:28-Lys-CTT-1-M4	GCC​CGG​CTA​GCT​CAG​TCG​GTA​GAG​CAT​G	28	tRF-5c	4.789248578	5.25259E-06
tRF-1:30-Lys-CTT-1-M2	GCC​CGG​CTA​GCT​CAG​TCG​GTA​GAG​CAT​GGG	30	tRF-5c	4.73980454	7.39006E-06
tRF-1:29-Val-CAC-2	GCT​TCT​GTA​GTG​TAG​TGG​TTA​TCA​CGT​TC	29	tRF-5c	4.738912076	4.63379E-05
tRF-1:31-Lys-CTT-2-M2	GCC​CGG​CTA​GCT​CAG​TCG​GTA​GAG​CAT​GAG​A	31	tRF-5c	4.709044346	1.71008E-06
tRF-1:31-Val-CAC-2	GCT​TCT​GTA​GTG​TAG​TGG​TTA​TCA​CGT​TCG​C	31	tRF-5c	4.673331537	1.83723E-05
tRF-+1:T32-Ala-AGC-8–2	GTT​TTC​CTT​CCT​GTC​CCG​TAC​GGT​TTT​TCT​TT	32	tRF-1	-5.667328485	0.002742496
tRF-30:43-Gln-CTG-1-M6	GACTCTGAATCCAG	14	tRF-2	-4.53559363	3.07914E-05
tRF-57:74-chrM.Phe-GAA	ATCACCCCATAAACACCA	18	tRF-3a	-4.302615301	0.00047173
tRF-55:76-Arg-ACG-1-M2	TCG​ACT​CCT​GGC​TGG​CTC​GCC​A	22	tRF-3b	-4.014776671	0.000369411
tRF-56:75-Gln-CTG-1-M2	AAA​TCT​CGG​TGG​AAC​CTC​CA	20	tRF-3b	-3.897122828	0.002044362
tRF-+1:T14-Gly-TCC-1	TGCGGTACCACTTT	14	tRF-1	-3.796561099	0.00050897
tRF-+1:T33-Gln-CTG-3	TTC​TGT​TTA​ATT​AGG​ACG​GCA​ATG​TTG​TGT​TTT	33	tRF-1	-3.539144569	0.006372188
tRF-+1:T15-Gly-TCC-1	TGCGGTACCACTTTT	15	tRF-1	-3.357284113	0.002185706
tRF-+1:T16-Gln-CTG-1–3	TTCATTTCTCTCCTTT	16	tRF-1	-3.348950695	0.002774635
tRF-+1:T32-Gly-CCC-1–2	AAA​GGG​TCT​TTT​TCA​CCC​CGC​TGT​TGC​TCT​TT	32	tRF-1	-3.248438763	0.033935869
tRF-+1:T17-Val-CAC-1	AAGTGGTTCCCGTTTTT	17	tRF-1	-3.114953848	0.001557773
tRF-53:69-chrM.Thr-TGT	TTTTTCCAAGGACACCA	17	tRF-3a	-3.097012264	0.00697687
tRF-+1:T32-Gln-CTG-3	TTC​TGT​TTA​ATT​AGG​ACG​GCA​ATG​TTG​TGT​TT	32	tRF-1	-3.063458661	0.021524971
tRF-+1:T28-His-GTG-1–3	ATG​TCG​TTA​GTC​TAG​GCT​GTC​AGC​TCT​T	28	tRF-1	-3.04172914	0.007708197
tRF-+1:T15-Arg-CCT-4	GCCTGTGACTTTTGT	15	tRF-1	-2.916120095	0.00712256

### Validation of Differentially Expressed tsRNAs by qRT-PCR

**FIGURE 4:**
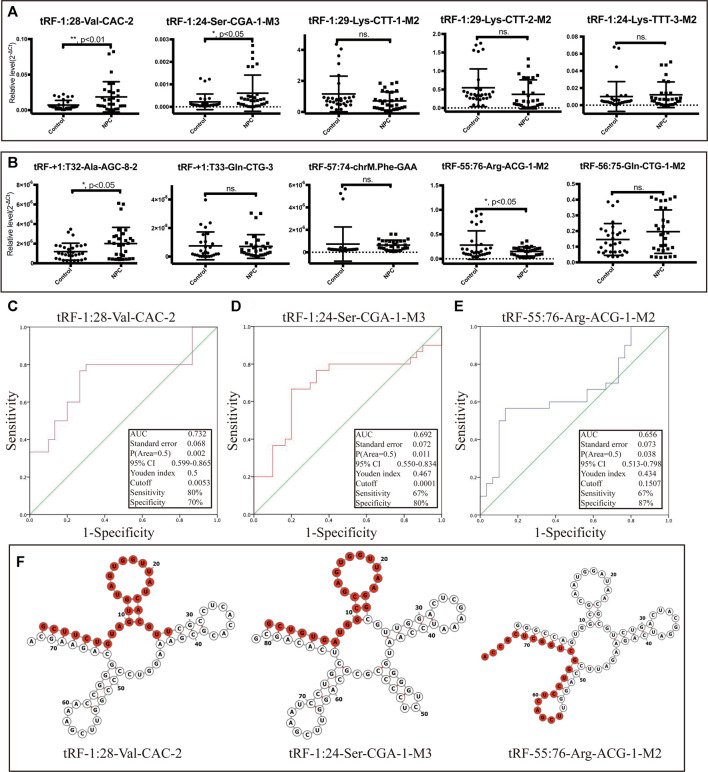
Dysregulation of selected tsRNAs in NPC by qRT-PCR. We selected 10 tsRNAs dysregulated in NPC for custom qRT-PCR. **(A)** Expression of 5 tsRNAs upregulated in NPC. **(B)** Expression of 5 tsRNAs downregulated in NPC as well. All data were analyzed using Student’s *t*-test (*p < 0.05, **p < 0.01). ROC curve analysis of **(C)** tRF-1:28-Val-CAC-2, **(D)** tRF-1:24-Ser-CGA-1-M3, and **(E)** tRF-55:76-Arg-ACG-1-M2. **(F)** Examples of tRNA or pre-tRNA structures of the three validated tsRNAs were depicted. The red nucleotides indicate the presence of the three validated tsRNA sequences.

The top five upregulated and top five downregulated tsRNAs that met specific primer design requirements were selected for verification from the differential sequencing results. The results show that in the selected five upregulated tsRNAs, the upregulated folds of tRF-1:28-Val-CAC-2 and tRF-1:24-Ser-CGA-1-M3 were consistent with the sequencing results (68.63- and 57.97-fold change, respectively) (Figure 4A). Among the five tsRNAs that were downregulated, only tRF-55:76-Arg-ACG-1-M2 met expectations with a 16.16-fold change (Figure 4B). Furthermore, these three validated tRFs were used for ROC analysis to evaluate the diagnosis efficiency in primary NPC. The results showed that the ROC-AUC for tRF-1:28-Val-CAC-2 was 0.732 [95% confidence interval (CI) (0.599–0.865)] for differentiating all NPC patients from the control, the best sensitivity and specificity were 80 and 70%, respectively (Figure 4C); tRF-1:24-Ser-CGA-1-M3 was 0.692 [95% confidence interval (CI) (0.550–0.834)] 67 and 80% (Figure 4D); tRF-55:76-Arg-ACG-1-M2 was 0.656 [95% confidence interval (CI) (0.513–0.798)] 67 and 87% (Figure 4E). In general, tRF-1:28-Val-CAC-2 has a relatively good ability to distinguish the primary NPC from the healthy controls. According to the cleavage position on the tsRNAs’ cloverleaf secondary structure, the validated tRFs were identified as tRF-5c, tRF-5b, and tRF-3b, respectively (Figure 4F).

### Potential Target Genes of Validated tRFs

**FIGURE 5:**
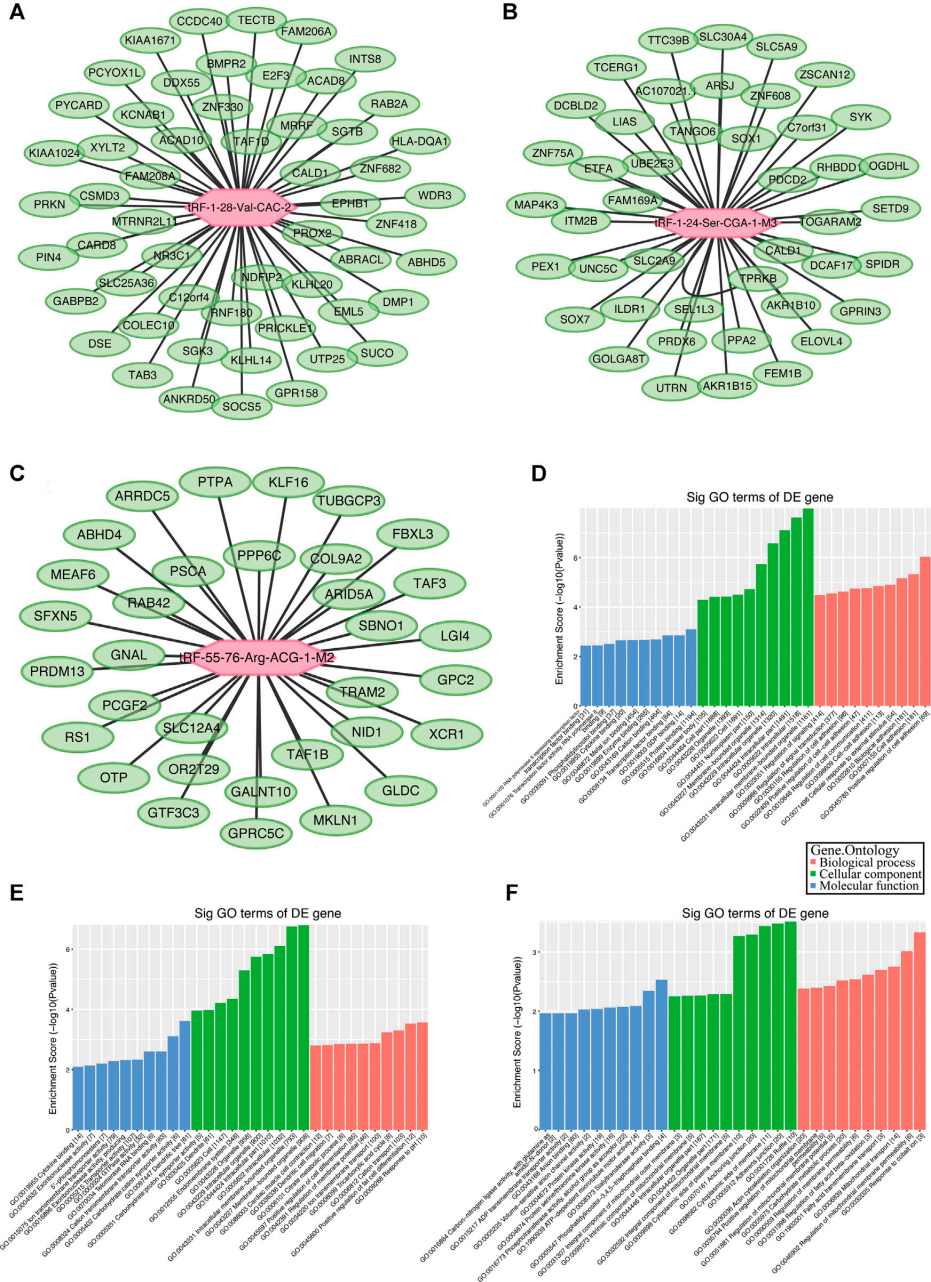
Gene ontology (GO) enrichment analyses for tRF-1:28-Val-CAC-2, tRF-1:24-Ser-CGA-1-M3, and tRF-55:76-Arg-ACG-1-M2. Putative target mRNA genes of **(A)** tRF-1:28-Val-CAC-2, **(B)** tRF-1:24-Ser-CGA-1-M3, and **(C)** tRF-55:76-Arg-ACG-1-M2 with context less than −0.4. **(D)** GO analysis showed that the target genes of tRF-1:28-Val-CAC-2 mainly participated in regulation of cell communication and signal transduction. **(E)** Analysis revealed that tRF-1:24-Ser-CGA-1-M3 was a key participant in regulation of cell differentiation. **(F)** Analysis revealed that tRF-55:76-Arg-ACG-1-M2 is particularly responsible for fatty acid transmembrane transport and regulation of membrane permeability.

Although the biological origin of tsRNAs is different from that of miRNAs, they are closely related to the effector proteins of miRNA, argonautes (AGO) 1, 3, and 4. Therefore, tsRNAs can play a role in miRNA-like RNA silencing by binding to AGO protein (Kumar et al., 2014). Using TargetScan and Miranda algorithms, we predicted potential target genes of the validated tRFs (tRF-1:28-Val-CAC-2, tRF-1:24-Ser-CGA-1-M3, and tRF-55:76-Arg-ACG-1-M2). Finally, we got 2093, 1,373, and 520 potential target genes separately (Supplementary File S2) and showed the genes with context < –0.4 (Figures 5A–C).

### The GO and KEGG Pathway Enrichment Analysis

**FIGURE 6:**
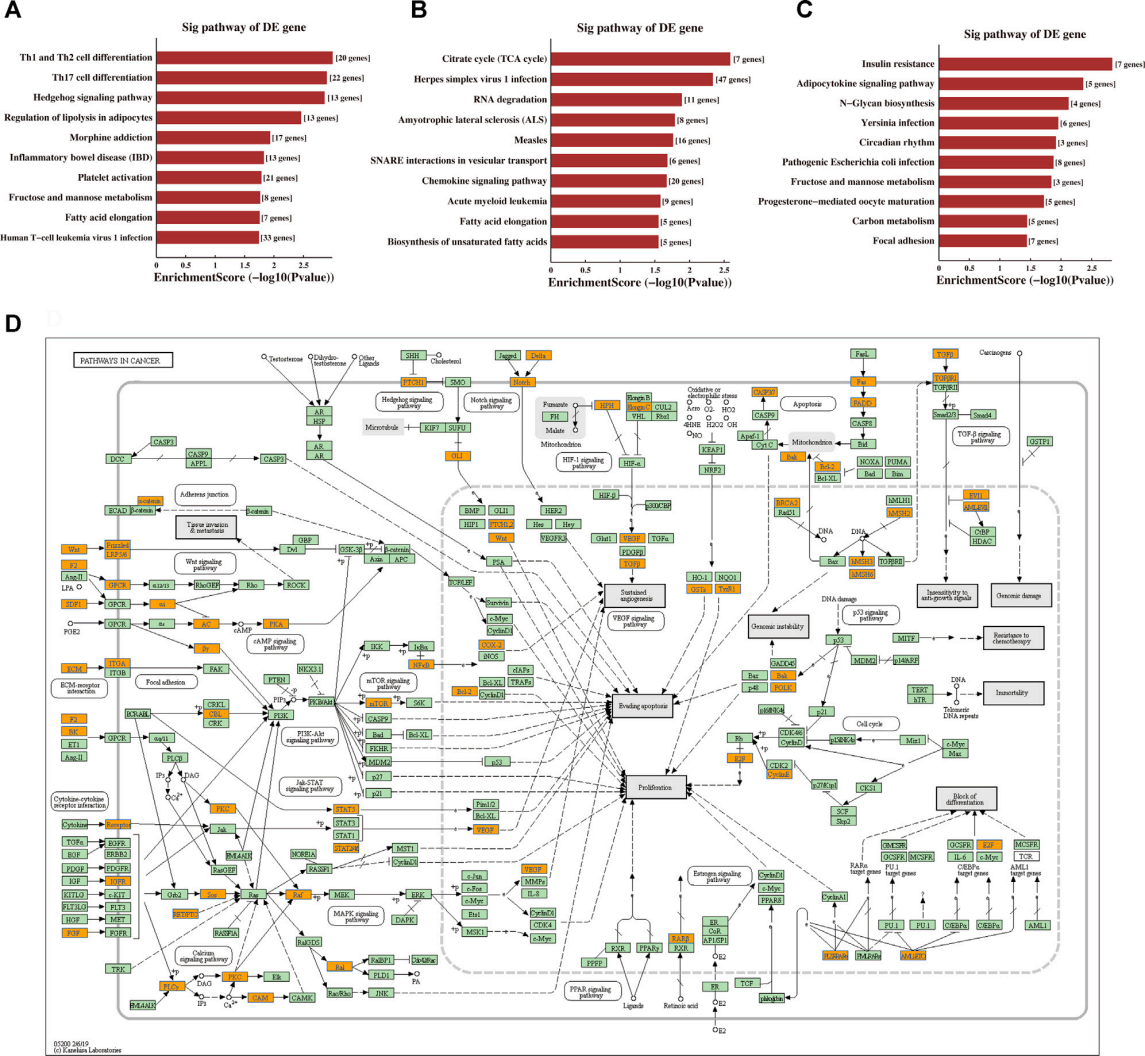
Prediction of signaling pathways of tRF-1:28-Val-CAC-2, tRF-1:24-Ser-CGA-1-M3, and tRF-55:76-Arg-ACG-1-M2. Kyoto Encyclopaedia of Genes and Genomes (KEGG) pathway analysis for **(A)** tRF-1:28-Val-CAC-2, **(B)** tRF-1:24-Ser-CGA-1-M3, and **(C)** tRF-55:76-Arg-ACG-1-M2-targeting genes. The vertical axis shows the annotated functions of the target genes. The horizontal axis shows the enrichment score (-log10 transformed *p*-value) and the gene number of each cluster, respectively. The KEGG pathways were considered significant if *p*-value < 0.05. Only the top 10 significantly enriched clusters are included. **(D)** Mapping of pathways in cancer. Orange-marked nodes correspond to target mRNAs from tRF-1:28-Val-CAC-2.

The BP, CC, and MF results obtained by GO enrichment analysis of target genes are displayed in Figures 5D–F (Supplementary File S3). The potential target genes of tRF-1:28-Val-CAC-2 are mainly located in the intracellular part and participate in cell communication, cell adhesion, and signal transduction regulation. Primary MFs of tRF-1:28-Val-CAC-2 were protein binding, transcription factor binding, and cytokine binding. For tRF-1:24-Ser-CGA-1-M3, the potential target genes are mainly situated in the membrane-bounded organelle, involved in response to pH, the tricarboxylic acid cycle, positive regulation of cell differentiation, and dendritic cell migration. Its primary MFs were GTPase activity, transporter activity, and cytokine binding. As for tRF-55:76-Arg-ACG-1-M2, potential target genes are usually found on the cytoplasmic side of the membrane and the mitochondrial membrane, and its primary molecular functions were ATP-dependent microtubule motor activity, protein kinase activity, and ADP transmembrane transporter activity. Furthermore, KEGG pathway enrichment analysis of three validated tRFs showed that 15, 17, and 14 pathways may be involved, respectively (Supplementary File S4). The GO terms and KEGG pathways were considered significant if *p*-value was < 0.05. The top 10 significant pathways are shown in Figures 6A–C. Among them, tRF-1:28-Val-CAC-2 plays a vital role in pathways in cancer (Figure 6D, although not in the top 10), including the predicted pathways: the Hedgehog signaling pathway (in the top 10) and the Wnt signaling pathway (not in the top 10). These tentative conclusions highlight the revolutionary significance of tsRNAs in primary NPC and enable the more in-depth investigation that is still needed to validate their mechanisms in the pathogenesis of NPC and to explore new diagnostic and therapeutic targets.

## Discussion

The prevalence of NPC is geographically limited, and EBV infection incidence is high (Chen et al., 2019). According to previous reports, tsRNAs can precisely reflect the disease’s state, even to the patient’s gender, race, and population (Telonis et al., 2015; Magee and Rigoutsos, 2020). Through high-throughput sequencing of tRFs and tiRNAs, we found 158 differentially expressed tRFs and tiRNAs in the primary NPC vs. healthy controls, which may be related to the pathogenesis of NPC. Among the ratio of subtype tRFs and tiRNAs, tRF-5c was over-expressed and tRF-1 was under-expressed in the NPC group. Although the type-number results did not show significant changes in the 3’tRF (tRF-3a and tRF-3b) abundance of NPC, the mean abundance of 3’tRF, especially the 22 nt 3’tRF, was relatively lower than that in the control group. Whether the dysfunction of 3’tRF in NPC can activate the transposons’ function, which will cause the tumors to lose epigenetic silence and promote tumorigenesis, is still worthy of further study (Schorn et al., 2017; Schorn and Martienssen, 2018). The subtype tRFs and tiRNAs against tRNA isodecoders are missing Arg-ACG, Gys-GCA, Met-CAT, Thr-TGT, and Trp-CCA in the NPC group. Altered tsRNA profiles can lead to metabolic disorders through epigenetic changes (Chen et al., 2016). The high metabolism of methionine (Met) (Wang et al., 2019a), arginine (Arg) (Poillet-Perez et al., 2018), cystine (Cys) (Badgley et al., 2020), threonine (Thr) (Zhang et al., 2018), and tryptophan (Trp) (Platten et al., 2019) was indispensable to the tumor development. Previous metabolomics studies of NPC have shown that l-alanine, serine, and glycine are significantly elevated in the serum of patients (Tang et al., 2011). In the study of Zheng et al., threonine, cysteine, and methionine were found to have an anticancer effect in nasopharyngeal carcinoma cells, and our study found that tsRNAs corresponding to these amino acids are missing (Zheng et al., 2019), which may mean that tsRNAs play an important regulatory role in the amino acid metabolism of NPC.

In subsequent studies, we selected ten significantly differentially expressed tsRNAs for verification. The results of qRT-PCT verified that two were upregulated (tRF-1:28-Val-CAC-2 and tRF-1:24-Ser-CGA-1-M3) and one was downregulated (tRF-55:76-Arg-ACG-1-M2) in line with the sequencing results. The ROC analysis evaluated the diagnostic ability of validated tRFs and found that tRF-1:28-Val-CAC-2 had better judgment ability (AUC = 0.732). In the future, it is still necessary to expand the samples and detect the level of tRFs and tiRNAs in serum at the same time to confirm its feasibility as a diagnostic indicator.

In general, tsRNAs mainly act on global translational activities through two pathways (Lin et al., 2014): AGO protein bound to tsRNAs cleaved from tRNAs and those tsRNAs that specifically bind to the mRNAs with partial complementarity and inhibit their translation (Zhang et al., 2015). Translation of some of the ribosomal proteins (RPs) and translational initiation or elongation factors (IEFs) is repressed by tsRNAs, which in turn suppresses the global translational activities (Kim et al., 2017; Luo et al., 2018). Previous studies have confirmed that tRFs and tiRNAs can bind to AGO proteins 1, 3, and 4, and then the AGO complexes can recognize and target mRNA to silence genes (Telonis et al., 2015). In predicting target genes, we found that tRF-1:28-VAL-CAC-2 was significantly correlated with AGO3 (context = –0.307), and ZDHHC20 was the mRNA with the strongest correlation (context = –0.714) with tRF-1:28-VAL-CAC-2. ZDHHC20 has been reported to enhance the antiviral activity by interferon-induced transmembrane protein 3 (IFITM3), which is worthy of further research on whether it can help people resist EBV infection (McMichael et al., 2017). At the same time, we found that zinc-finger protein 418 (ZNF418, context = -0.692), as a TOP3 prediction target gene, has been widely studied in tumors and plays an antitumor role in gastric cancer (Hui et al., 2018), liver cancer (Wang et al., 2019b), and esophageal cancer (Pu et al., 2017). ZNF418, a DNA methylation regulator, is a transcriptional repressor, negatively affecting the MAPK signaling pathway, which plays an important role in NPC progression, chemotherapy resistance, and radiotherapy resistance (Zhang et al., 2019; Zheng et al., 2020).

Furthermore, the KEGG pathway enrichment analysis of tRF-1:28-Val-CAC-2 was enriched in pathways in cancer, which included the Hedgehog signaling pathway with EVI1, BCL2, and PKA (Yip et al., 2006; Lu et al., 2019) and the Wnt signaling pathway (not in the top 10) with Frizzled, Daam1, and NFAT (Ren et al., 2015). Besides, missense mutations in the Hedgehog signaling pathway and the Wnt signaling pathway (Tu et al., 2018) were detected in 12 pairs of EBV + NPC specimens. Previous *in vitro* experiments have confirmed that EBV-infected epithelial cells activate the Hedgehog signaling pathway to cause stem cell phenotype (Port et al., 2013). The Wnt signaling pathway also plays an essential role in the maintenance of cancer stem cells (Chan et al., 2020). Also, it is possible to target Th1 and Th2 cell differentiation, Th17 cell differentiation, and the T cell receptor signaling pathway (not in the top 10) with CTLA4, CD28, NF-kB, and GM-CSF to affect the changes of the immune microenvironment. In the future, our experimental plan is to verify the mechanisms of tRF-1:28-Val-CAC-2 in the progression of NPC. The other two tRFs (tRF-1:24-Ser-CGA-1-M3 and tRF-55:76-Arg-ACG-1-M2) mainly participated in regulating cell differentiation, immune cell migration, and protein kinase activity. The KEGG pathway enrichment analysis of the other two tRFs mainly involves the citrate cycle (TCA cycle), the chemokine signaling pathway, autophagy, the PI3K-Akt signaling pathway, and the AMPK signaling pathway, and they are also worth exploring.

Although more and more researchers have begun to pay attention to the research on tsRNAs, there is still no unified standard for their classification, nomenclature, and target prediction method. Various tRF profiling methods have distinct advantages and disadvantages (MINTmap, tDRmapper, tRFdb, and tRFfinder) (Loher et al., 2017; Xie et al., 2020). The limitation of this article is that the use of bowtie software mapping small RNA data cannot distinguish i-tRF (which overlaps with tRF-2), and the target prediction method of using a miRNA-like pattern is relatively limited. For tsRNA target prediction, miRNA-like prediction (Mo et al., 2019) and tRF–mRNA correlation prediction (Telonis et al., 2019) are commonly used, and more accurate prediction requires that experiments to determine be designed, such as cross-linking ligation and sequencing of hybrids (CLASH) and covalent ligation of endogenous AGO-bound RNAs [(CLEAR)-CLIP] (Xiao et al., 2021). Therefore, it is necessary to establish more effective research methods in order to better identify and understand the biological function of tsRNAs.

More and more cases of dysregulation of tsRNAs in various cancers have been reported in recent years, suggesting that tsRNAs indeed play an important role in the development of tumors. Our study provides the abnormal expression profile of tRFs and tiRNAs in primary NPC for the first time. tsRNAs have also been found to be stable in peripheral circulation and have a higher abundance than miRNAs. With the increasing application of circulating nucleic acids in serum and other body fluids for cancer screening and prognosis (such as liquid biopsy), the application of tsRNAs as cancer biomarkers has obvious advantages (Zhu et al., 2019b). These identified tsRNAs may become a new class of biological diagnostic and prognostic indicators, even essential targets for NPC treatment in the future, and its specific carcinogenic mechanism is worthy of further study.

## GenBank

The sequence data for this article are submitted to GEO with the Accession No. GSE159746 (https://www.ncbi.nlm.nih.gov/geo/query/acc.cgi?acc=GSE159746).

## Data Availability

The datasets presented in this study can be found in online repositories. The names of the repository/repositories and accession number(s) can be found below: https://www.ncbi.nlm.nih.gov/, GSE159746.
